# Petit-spot as definitive evidence for partial melting in the asthenosphere caused by CO_2_

**DOI:** 10.1038/ncomms14302

**Published:** 2017-02-02

**Authors:** Shiki Machida, Tetsu Kogiso, Naoto Hirano

**Affiliations:** 1 Research and Development Center for Submarine Resources, Japan Agency for Marine-Earth Science and Technology, Natsushima-cho 2-15, Yokosuka, Kanagawa 237-0061, Japan; 2 School of Engineering, The University of Tokyo, 7-3-1 Hongo, Bunkyo, Tokyo 113-8654, Japan; 3 Graduate School of Human and Environmental Studies, Kyoto University, Yoshida-nihonmatsu, Sakyo, Kyoto 606-8501, Japan; 4 Center for Northeast Asian Studies, Tohoku University, Kawauchi 41, Aoba-ku, Sendai 980–8576, Japan

## Abstract

The deep carbon cycle plays an important role on the chemical differentiation and physical properties of the Earth's mantle. Especially in the asthenosphere, seismic low-velocity and high electrical conductivity due to carbon dioxide (CO_2_)-induced partial melting are expected but not directly observed. Here we discuss the experimental results relevant to the genesis of primitive CO_2_-rich alkali magma forming petit-spot volcanoes at the deformation front of the outer rise of the northwestern Pacific plate. The results suggest that primitive melt last equilibrated with depleted peridotite at 1.8–2.1 GPa and 1,280–1,290 °C. Although the equilibration pressure corresponds to the pressure of the lower lithosphere, by considering an equilibration temperature higher than the solidus in the volatile–peridotite system along with the temperature of the lower lithosphere, we conclude that CO_2_-rich silicate melt is always produced in the asthenosphere. The melt subsequently ascends into and equilibrates with the lower lithosphere before eruption.

## Abstract (web summary)

The genesis of primitive CO_2_-rich alkali magma at petit-spot volcanoes is still poorly understood. Here, the authors perform high pressure and temperature experiments to show that the CO_2_-rich melt is derived from the asthenosphere.

The nature of the seismic low-velocity zone in the upper mantle, the asthenosphere, is a matter of debate. Hirschmann^1^ showed that the peridotite–CO_2_–H_2_O system melts and produces carbonatite or CO_2_-rich silicate melt at the asthenosphere under normal thermal gradient. Subsequently, the existence of CO_2_- and H_2_O-rich melt in the asthenosphere was suggested by experimental determination of electrical conductivity of such melt^2^; however, we have no direct observations or evidence of melt yet. Petit-spot volcanism^3^, which is plate deformation-induced eruption of alkali magma forming diminutive volcanoes on the oceanic plate, is expected as the evidence for melt in the asthenosphere because of the following observations. No upwelling of hot, deep mantle was observed by seismic tomography^4^, indicating that petit-spot did not originated from an active mantle plume^3^. Okumura and Hirano^5^ determined 10% of CO_2_ and 1% of H_2_O in the primary petit-spot magma on the basis of measurement of CO_2_ and H_2_O content in the glassy rinds of lavas using infrared spectroscopy and back calculations of primary content considering magma degassing along the path of ascending magma and the vesicularity of lava. In addition, several petit-spot volcanic fields have been reported in the northwestern Pacific^3^,^6^,^7^, the ocean-ward slope of the Tonga Trench^8^, the Chile Trench^9^ and the Sunda Trench^10^, the Basin and Range Province of North America^11^, offshore southern Greenland^12^ and the Santa Rosa accretionary complex in Costa Rica^13^. These observations suggest that petit-spot volcanism is ubiquitous phenomenon in the regions of plate flexure owing to oceanic plate subduction^3^,^6^,^7^,^8^,^9^,^10^ and glacial melting^12^. Therefore, if magma originates in the asthenosphere, as originally proposed by Hirano *et al*.^3^, petit-spot volcanism should provide critical insight into melt production in the asthenosphere based on the high amount of CO_2_ in the melt and the ubiquitous distribution of volcanic fields. Obviously, a comprehensive model for petit-spot volcanism from magma genesis to eruption is desirable.

A model for the eruption of petit-spot volcanoes was first proposed by Hirano *et al*.^3^ The authors proposed that a petit-spot volcano forms by exuding magma that originates in the upper asthenosphere and passes through the lithosphere in response to plate flexure during the formation of the outer rise. In addition to this basic model, Yamamoto *et al*.^14^, based on peridotite xenoliths, suggested that the formation of melt ponds before eruption at the lithosphere–asthenosphere boundary (LAB) is needed to explain the localized hot geotherm of the petit-spot volcanoes. The ponding is caused by the horizontal melt migration against the plate motion beneath the LAB owing to the pressure gradient that is induced by the excess topography of the outer rise, which is the difference in depth between the shallow seafloor at the top of the outer rise and deep normal seafloor^14^. Machida *et al*.^7^ further ascertained that the position of the eruption of magma in a petit-spot volcanic field temporally migrates opposite to the direction of the movement of the Pacific plate, accompanying gradual change of the erupted lava geochemistry. These observations were explained by a new eruption model^7^ that considered a petit-spot volcanic field to correspond to an isolated melt pond at the LAB defined by Yamamoto *et al*.^14^ The melt pond is dragged by the plate motion, while it is being constantly supplied with new magma (magma mixing) and moves slightly slower than the plate and repeatedly induces melt eruption owing to plate flexure^7^.

Nevertheless, three critical problems need to be understood in petit-spot genesis. First, the ‘plate-flexure model'^3^,^7^,^14^ explains the eruption mechanism reasonably well. However, the model requires the existence of melt in the asthenosphere. On the viewpoint, second, if petit-spot genesis is attributed to the formation of the outer rise based on the ‘plate-flexure model', volcanoes should be commonly distributed along the outer rise. However, they are not. For example, Hirano *et al*.^3^ and Machida *et al*.^7^ showed that the three petit-spot volcanic fields in the northwestern Pacific are not continuous along the outer rise. This observation suggests that the melting processes, not only plate-flexure, constrain the locus of petit-spot magmatism. Third, to understand the melting processes, our previous geochemical studies have shown that alkali lava from petit-spot volcanoes have high concentrations of incompatible trace elements indicating extreme enrichment in highly incompatible elements, (for example, Rb, Ba, U, Th and Nb) and light rare earth elements^3^,^7^, and have extreme enriched mantle 1-like Sr–Nd–Pb isotopic compositions^7^,^15^. Machida *et al*.^15^ thus proposed that melting of small blobs of recycled ancient plate materials (small-scale heterogeneity) in the upper mantle produces petit-spot magmas. This model is critical but the melting conditions and lithology of the source material are debated. Because all of the previous models that demonstrate the origin of petit-spot volcanism^3^,^7^,^14^,^15^ do not constrain the melting processes in the asthenosphere, we clarify whether or not the petit-spot melt is generated in the asthenosphere in this study.

To define the magma genesis of petit-spot volcanoes, the independent determination of temperature and pressure conditions for magma production is required. We thus performed melting experiments to define the melting phase relations of petit-spot primary magmas. We report the results of high-pressure melting experiments for basalts from the two youngest knolls (erupted between 0.05 and 1 Ma^3^) situated in the flexed region of the northwestern Pacific plate, while considering the phase relations of the CO_2_–H_2_O–melt system. Our experiments aim to constrain the temperature and pressure conditions of melt segregation and the source lithology. Thus, we map the liquidus mineralogy to locate the melt saturation with two or three phases (the multiple saturation points) to constrain the last equilibration pressures and temperatures of primary magmas before ascending to the seafloor.

## Results

### Accuracy evaluation of experiments

**Figure 1:**
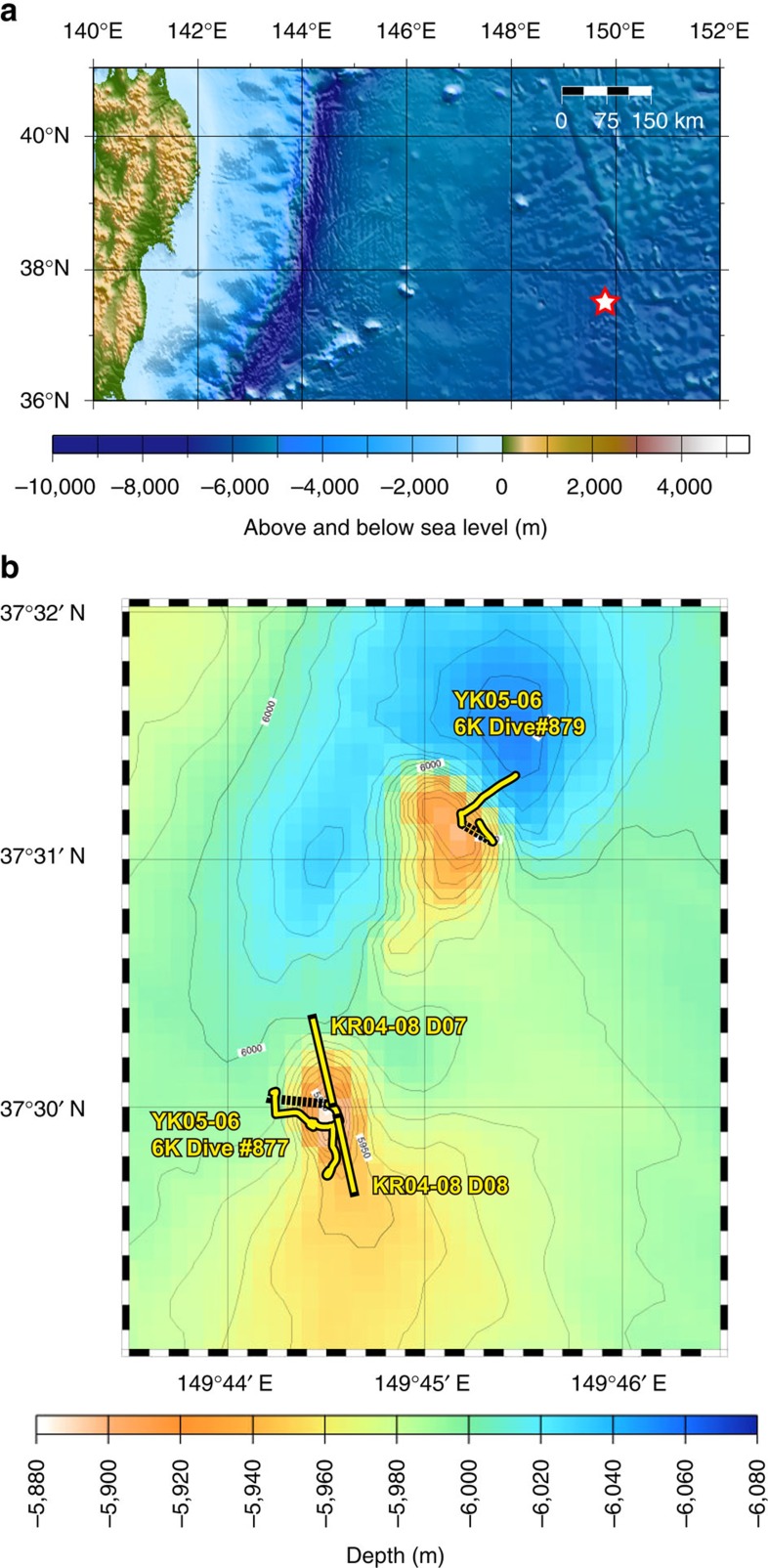
Bathymetric maps for northwestern Pacific showing the position of the petit-spot volcanoes investigated in this study. Bathymetric data are from ETOPO1 (NOAA National Geophysical Data Center, http://www.ngdc.noaa.gov/) for **a** and collected by multi narrow beam survey for **b** (ref. 7). Open red star for **a** nearly corresponds to the region shown in **b**. Yellow thick lines for **b** mark the position of the survey lines around the sampling sites^7^.

We conducted high pressure and temperature experiments for primary basalts from the two youngest knolls of petit-spot in the northwestern Pacific plate (Fig. 1). The H_2_O content of the starting materials (3.0–4.2 wt%; Table 1) is higher than the estimated initial H_2_O content of petit-spot melt^5^ (H_2_O content of melt at the saturation point at 0.16–0.19 GPa before bubble formation; ∼1.0 wt%). This is probably owing to moisture absorption by alkali carbonates and magnesium oxide in the starting mixtures during mixing of the reagent powders (see Methods). However, we expect that H_2_O release from the melt occurs during high-pressure magma ascend relative to the H_2_O-saturated pressure. Hence, we think that the starting materials reasonably represent the primary H_2_O content of the melt before the H_2_O release.

**Figure 2:**
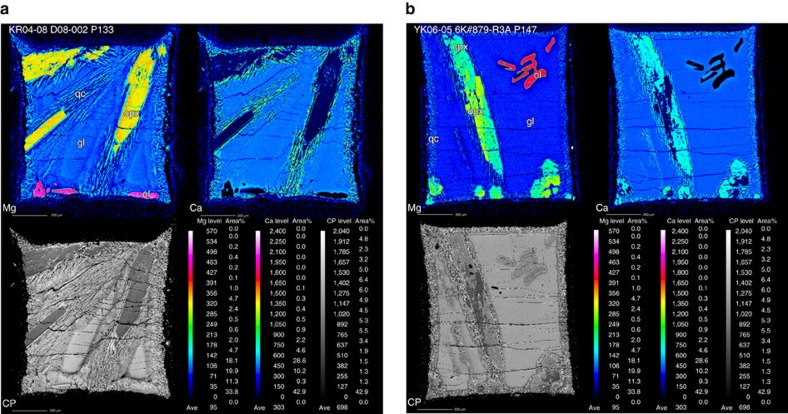
EPMA mapping images. Magnesium (Mg) and calcium (Ca) and a compositional image in backscattered electron mode (CP) for representative run products for the sample KR04-08 D08-002 (**a**) and YK06-05 6K#879-R3A (**b**) are shown. Ol: olivine, cpx: clinopyroxene, opx: orthopyroxene, gl: glass, qc: quenched crystals.

All of run products comprise glass and the zone of quenched crystals. In the case of below the liquidus, solid phases were observed (Fig. 2). Solid phases are commonly >50 μm in diameter except for just below the liquidus. The quenched crystals are grown at the contact with the graphite capsule and each solid phase and then surround glass. Therefore, it is clear that the melt phase was solidified as glass and the quenched crystals. The *∑R*^2^ range from 0.210 to 1.580 (Supplementary Data 1), indicating reasonable mass balances between the analysed solid and melt phases and starting bulk composition, along with the other criteria suggests approach to equilibrium (see Methods; Supplementary Data 1). The determined compositions of the phases are given in the Supplementary Data 2. The magnesium (Mg)-numbers (Mg#) (Mg/(Mg+Fe) in mole percent) of near-liquidus olivine and orthopyroxene (0.86) are slightly lower than mantle values (0.90) (Supplementary Data 1). In our experiments, we used Pt–graphite capsules. Médard *et al*.^16^ showed that the *f*O_2_ in the Pt–graphite capsule is 0.8 log units below the CCO (graphite–carbon dioxide) buffer or 1.4 log units above the IW (iron–wüstite) buffer, which is lower than the actual melting conditions in the mantle (+2 ΔIW log units)^17^. In the reduced conditions of the Pt–graphite capsules, the Mg# likely decreases because Fe^3+^ in the starting materials (Fe_2_O_3_) reduced to Fe^2+^ (FeO). Therefore, the lower *f*O_2_ is likely the cause of the lower olivine Mg# than that of peridotite. However, the phase relations depend more on the SiO_2_ activity in the melt than the Fe–Mg exchange^18^. We thus consider that the differences in the olivine Mg# to minimally affect our results.

### The P–T phase relations for primitive magma

**Figure 3:**
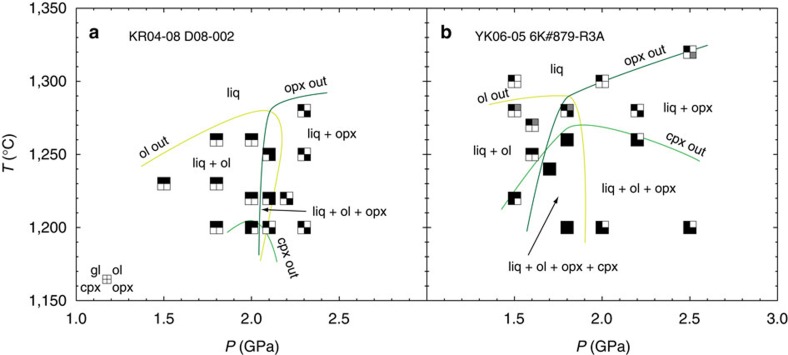
Melting phase relations of the primary petit-spot basalts. Results for the sample KR04-08 D08-002 and YK06-05 6K#879-R3A are shown in **a** and **b**, respectively. Stable phases are shown by the filled squares. Grey squares denote the expected crystalline phases on the basis of mass balance calculation for the low residual sums of squares (*∑R*^2^) (<2.000; Supplementary Data 1). Ol: olivine, cpx: clinopyroxene, opx: orthopyroxene, liq: glass and the quenched crystals.

D08-002 has olivine (ol) on the liquidus at pressures lower than 2.1 GPa, whereas orthopyroxene (opx) is the liquidus phase at higher pressures (Fig. 3). Liquidus temperatures of approximately 1,270, 1,280 and 1,290 °C were respectively estimated on the basis of the change in the proportions of ol and opx with increasing temperature on experiments at 1.8 GPa, 2.0 GPa and 2.3 GPa. The melt is cosaturated with ol and opx at 2.1 GPa and 1,250 and 1,220 °C. Olivine disappears at lower than approximately 1,210 °C at 2.1 GPa. Clinopyroxene (cpx) is found only at 2.0 GPa and 1,200 °C with melt and ol. We thus conclude that D08-002 liquid is multiply saturated with ol and opx at 2.1 GPa and 1,280 °C, and cpx joins in at approximately 80 °C below the liquidus.

Ol or opx is respectively the liquidus phase at pressures ≤1.8 GPa or ≥1.9 GPa for 6K#879-R3A (Fig. 3). The melt is cosaturated with ol, cpx, and opx at between 1.7 GPa and 1.8 GPa and 1,260 °C or lower. In the case of the experiment at 2.5 GPa and 1,320 °C, mass balance calculations using the compositions of the observed melt phases (glass and the quenched crystals on polished section of the run product) shows high *∑R*^2^. However, recalculation adding opx (composition same as the opx observed in the experiment at 2.5 GPa and 1,300 °C) to the solid phases decreased the *∑R*^2^ to less than 1 (Supplementary Data 1). Therefore, we consider that opx was present at 2.5 GPa and 1,320 °C even though it was not observed in the polished section. Although ol was not observed in the experiments at 1.5 GPa and 1,280 °C, 1.6 GPa and 1,270 °C and 1.8 GPa and 1,280 °C, the calculation after adding ol (same composition as the ol observed in the experiment at 1.6 GPa and 1,250 °C for the first two experiments and ol observed in the experiment at 1.8 GPa and 1,260 °C for the latter) to solid phase decreased the *∑R*^2^ to <1 (Supplementary Data 1). Thus, 6K#879-R3A liquid is multiply saturated with ol and opx at 1.8 GPa and 1,290 °C, and cpx joins in at approximately 20 °C below the liquidus. Two phase stability fields, ol–cpx at low pressure and two pyroxenes at high pressure, were also observed.

## Discussion

**Figure 4:**
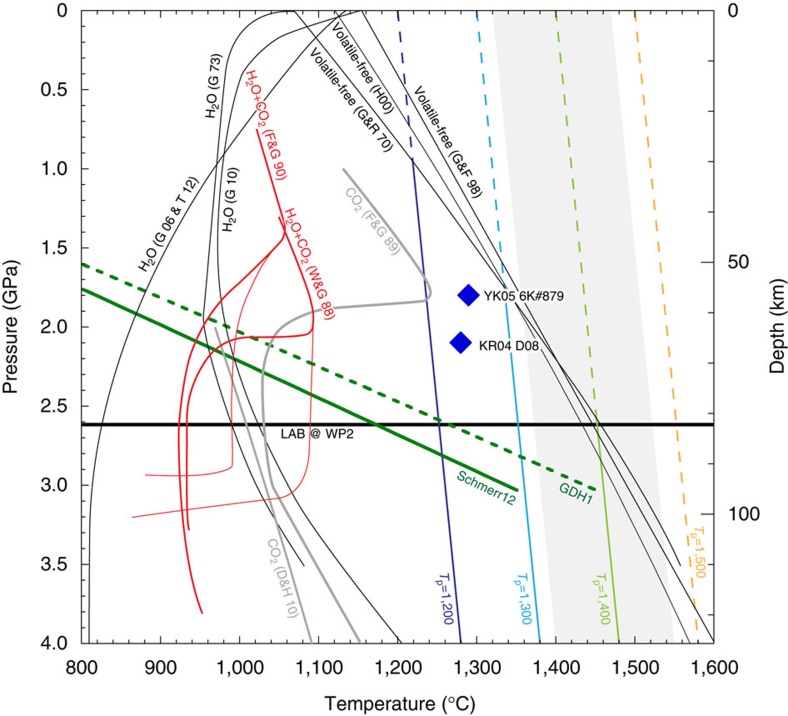
Diagram for the last equilibration temperature and pressure of the primary petit-spot magma. Our results are compared with the solidus distribution determined by melting experiments in the volatile–peridotite system. Black solid lines mark the solidus for volatile-free peridotite^17^,^40^,^41^ and H_2_O–peridotite^42^,^43^,^44^,^45^ system. Grey solid lines mark the solidus in the CO_2_–peridotite^46^,^47^ system. Red lines denote the solidus in the peridotite–CO_2_–H_2_O (refs 23, 24) system. The green thick line and dashed line respectively denote the thermal structure of the 130 Ma plate estimated on the basis of plate model assuming the variables proposed by Schmerr^21^ (Schmerr 12) and Stein and Stein^22^ (GDH1). The black thick horizontal line marks the depth of the lithosphere–asthenosphere boundary (LAB) for the northwest Pacific (82 km depth at WP2 (ref. 20)). The blue, light blue, light green and orange lines are adiabats corresponding to the given mantle potential temperature (*T*_P_) for 1,200 °C, 1,300 °C, 1,400 °C and 1,500 °C, respectively, proposed by Dalton *et al*.^19^ The grey zone shows the global variation of the mantle potential temperature beneath the mid-ocean ridge^19^.

On the basis of our experiments, the multiple saturation point of the primary petit-spot melt is at 1.8–2.1 GPa and 1,280–1,290 °C (Fig. 4), indicating that the petit-spot magma last equilibrated with harzburgite ∼60 km deep under slightly lower temperature than the adiabat of the mantle potential temperature (MPT) of 1,250 °C (ref. 19). This is shallower than the depth of the LAB for the northwest Pacific (82 km depth at WP2 (ref. 20)), suggesting that the last equilibrium depth of the petit-spot magma is within the lower lithosphere. If the estimated last equilibration pressure suggests segregation depth of the primary petit-spot melt from the solid phases at the lower lithosphere, it is reasonable to argue that the primary petit-spot magma with a high amount of CO_2_ originated in the asthenosphere, as shown in the following.

Assuming the plate model^21^,^22^, we calculated the thermal structure of the 135 Ma lithosphere beneath the petit-spot volcanoes considered in our experiments (Fig. 4). Figure 4 shows that the estimated temperature below the lithosphere at 3.0 GPa corresponds to the adiabat of the low-temperature side of the global variation in the MPT along the mid-ocean ridge (1,320 °C)^19^, and is significantly higher than the solidus temperature of fertile peridotite with CO_2_ and H_2_O (refs 23, 24). Thus, if CO_2_ or carbonate exists in the asthenosphere, melt production is anticipated. This line of discussion was simulated previously in the peridotite–CO_2_–H_2_O system^1^. Based on the calculations by Hirschmann^1^, the CO_2_-rich silicate melt is likely stable in the asthenosphere at the MPT between 1,300 to 1,400 °C. Sakamaki *et al*.^25^ experimentally observed the low melt viscosity and the large difference between the densities of melt and ambient olivine, that is, high melt mobility, in the range of 120–150 km; thus, they proposed that ascending melt in the asthenosphere should accumulate at the LAB. As mentioned in the introduction, the accumulated melt at the LAB further migrates horizontally owing to the pressure gradient induced by the formation of the outer rise^14^, forming isolated melt ponds^7^. Therefore, we propose the following model for the processes of petit-spot volcanism to explain the estimated last equilibration pressure and temperature of the primary melt on the basis of our experiments. CO_2_-rich silicate melt is commonly produced in the asthenosphere^26^ along the adiabat of the low-temperature side of the global variation of the MPT along the mid-ocean ridge (∼1,320 °C) and accumulates to form melt ponds at LAB, followed by equilibration with harzburgite at the lower lithosphere before eruption.

A rebuttal case for the genesis of the primary petit-spot magma is the *in situ* melting of carbonaceous peridotite or of a normal (non-metasomatized) peridotite with flux of CO_2_ (and H_2_O) fluid in the lower lithosphere. The thermal structure model for the 135 Ma lithosphere, such as GDH1 (ref. 22; Fig. 4), shows that the geotherm intersects the solidi for the peridotite–CO_2_–H_2_O system^23^,^24^ (melting of the peridotite–CO_2_–H_2_O system is possible) at ∼2 GPa and 1,000–1,100 °C. However, this temperature is significantly lower than that of the primary petit-spot melt in our experiments. Although a heat source is necessary to cause in-situ melting at the temperature of the last equilibration of the primary petit-spot melt, the upwelling of hot deep mantle is not observed by seismic tomography beneath the petit-spot volcanoes^4^. Therefore, in-situ melting of the lower lithosphere is not probable.

To explain our results, especially of the last equilibration temperature of the petit-spot primary magma, CO_2_-rich silicate melt has to be produced in the asthenosphere because of the existence of CO_2_-rich fluid or carbonate. However, our experiments also suggest that the melt segregation from solid phases may occur at the lower lithosphere. Therefore, we have to connect the melt pond at the LAB^7^,^14^ and the melt that equilibrated in the lower lithosphere. At the deformation front of the outer rise (examined in this study), the lower lithosphere experiences extensional stress owing to the concave bending of the plate^11^. Then, it is reasonable to argue that ascending occurs faster than the cooling of melt by the ambient lithologies. Moreover, the stress field changes from extensional to compressional at midlithospheric depths^11^, probably corresponding to the slightly shallower than the last equilibration depth (approximately 60 km, equivalent to 2 GPa) of the primary petit-spot magma obtained in this study. Therefore, we interpret the last equilibration depth as the depth where melt ascending stops or slows owing to the stress rotation in the lithosphere^11^. We thus conclude that the original eruption model for petit-spot^3^,^7^, considering direct exuding of the melt through the lithosphere, needs to be slightly modified; that is, (a) CO_2_–and H_2_O–rich melt ponding at the LAB ascends the overlying lithosphere owing to plate flexure, (b) the ascending melt equilibrates with harzburgite at approximately 1,280 °C and 60 km depth that corresponds to the base of the elastic lithosphere and finally (c) melt erupts on the seafloor. As the support for b, melt entrapment at the lower lithosphere can explain the localized anomalies of high electrical conductivity at ∼60 km in the lithosphere^27^ and the extremely high geotherm^14^ just beneath the petit-spot volcanoes. To constrain c, rapid lava eruption is suggested from the presence of xenocrysts and xenoliths, which represent lithospheric lithologies in petit-spot lavas^3^. The deepest peridotitic xenolith (from ∼45 km depth (1.3 GPa)^28^) suggests that melt ascends from the lower lithosphere before eruption on the seafloor. Detailed melt ascending processes through the lithosphere will be investigated in the future.

The principal constraint on the petit-spot origin revealed in this report and our previous studies^7^,^14^ is the role of CO_2_-fluid or carbonate in partial melting in the asthenosphere. Melting of small blobs of recycled ancient plate materials (small-scale heterogeneities) in the upper mantle produces petit-spot magmas^15^, thus, recycled plate materials could be the source of the CO_2_-fluid or carbonate in the asthenosphere. Hence, blobs of CO_2_-fluid- or carbonate-rich material in the asthenosphere is expected to constrain the loci of petit-spot magmatism (the second critical problem in petit-spot genesis, as pointed out in the introduction). Future detailed geochemical and petrological investigations of petit-spot lavas will provide insights of the linkage between the lithology of the seismic low-velocity layer and global carbon recycling.

## Methods

### Sample selection

Samples D08-002 and 6K#879-R3A were selected as representative of petit-spot basalts. They were collected from two isolated knolls during cruises KR04-08 of *R/V Kairei* (by dredge) and YK06-05 of *R/V Yokosuka* (by dive of the *Shinkai 6500* submersible) (Fig. 1). These knolls are situated in a petit-spot volcanic field ∼600 km ESE of the Japan Trench and correspond to the deformation front of the outer rise formation (Fig. 1). These volcanoes on the Cretaceous Pacific plate formed approximately 136 Ma^7^,^29^,^30^. Samples D08-002 and 6K#879-R3A are classified as basanite or trachybasalt^3^,^7^ and into Group 3 basalt (defined by Machida *et al*.^7^) with negative Zr and Hf anomalies on the spidergram for trace elements normalized to primitive mantle. They are likely to be closer in composition to the primary melt because their FeO*/MgO ratios are close to unity (1.05 and 1.53) and have high Cr and Ni contents (>90 p.p.m.) (Table 1).

### Preparation of starting materials

The original bulk-rock compositions of samples D08-002 and 6K#879-R3A (Table 1) equilibrates with Fo (that is, 100Mg/(Mg+Fe) in mole per cent)=86 and 81 olivine. This observation indicates that composition of petit-spot magma was changed from primary Mg-rich magma, which equilibrates with mantle, owing to crystal fractionation before eruption. Therefore, for the precise experiment, the primary composition of starting material has to be reconstructed from the original composition by taking into account olivine fractionation. We consider that olivine-bearing lithology should exist in the source, even if contributions of recycled materials are expected^31^. Furthermore, involvement of recycled materials into the magma source would not significantly affect the Fo content of olivine in the source^31^,^32^,^33^. Thus, the major element compositions of primary melt equilibrated with Fo=90 olivine (Table 1) have been reconstructed from the original compositions of samples D08-002 and 6K#879-R3A using the olivine maximum-fractionation model^32^,^34^,^35^. We also consider that a primary magma includes approximately 10% CO_2_ and more than 1% H_2_O. Then, starting materials were prepared at Kyoto University by mixing pre-dried reagents of oxides, hydroxides, phosphate and carbonates to represent the major elements, CO_2_ and H_2_O composition of the primary magma for each petit-spot volcano (Table 1). Powders of MgCO_3_, Mg(OH)_2_ and MnO were dried at 110 °C for more than 1 day. Powders of SiO_2_, TiO_2_, Al_2_O_3_, Fe_2_O_3_ and Ca_3_(PO_4_)_2_ were dried in a muffle furnace at 500 °C overnight. Powders of CaCO_3_, K_2_CO_3_ and Na_2_CO_3_ were dried in a muffle furnace at 300 °C overnight. MgO powder was dried in a muffle furnace at 1,000 °C for >4 h. The reagent mixture was then carefully ground in an agate mortar under ethanol for >1 h.

The bulk H_2_O content was measured by an ADP-512 Karl Fisher moisture titrator at the Earthquake Research Institute, University of Tokyo. After heating at 120 °C to remove any moisture absorbed from air, each powdered sample (∼100 mg) was heated at 1,000 °C for 15–20 min until no further release of moisture could be observed. The bulk CO_2_ content was determined on the basis of the total carbon in the starting materials, which was analysed using a CHNS (carbon, hydrogen, nitrogen and sulphur) analyzer (Vario EL III; Elementar Co. Ltd.) at the Japan Coast Guard Academy. Starting materials weighing ∼20 mg were used in the CHNS analysis at 1,150 °C and 90 s.

### Melting experiments

Melting experiments were conducted using a Boyd–England-type 1/2-in-diameter piston–cylinder apparatus (PG-100; C & T Factory) at Kyoto University. Starting material of ∼1 mm diameter and height was packed into graphite capsule, which was subsequently sealed in platinum (Pt) capsule. The Pt capsule was crimped and welded shut using a carbon arc welder. The sealed capsule was positioned on the centre of the 31-mm furnace assembly of MgO inner pieces, graphite heater, and Pyrex glass and talc sleeves from inside to the outside. A steel plug with a pyrophyllite sleeve was placed on top of the furnace assembly. The pressure was calibrated at 900 °C using the quartz to coesite transformation at 3.0 GPa (ref. 36), and at 1,400 °C using the protoenstatie to high-temperature orthoenstatite transition at 0.85 GPa (ref. 37). The temperature was monitored with a Pt–Pt_87_Rh_13_ thermocouple and controlled using a digital program controller (KP100c; CHINO). The thermal gradient in the assembly was investigated at 2.0 GPa and at 1,250 °C using an enstatite–diopside mixed powder and the two-pyroxene geothermometer^38^. The average temperature in the sample position (*n*=25) was 1,258 °C (s.d.=43 °C), which indicates the limited thermal gradient. The difference between the average temperature and the temperature reading of the thermocouple is within the error of geothermometer. The experiments were conducted at 1.5–2.5 GPa and 1,200–1,320 °C (Supplementary Data 1). The starting material was melted under the target pressure at 1,400 °C for 2 h and then the melt and solid phases were equilibrated at the target temperature at constant pressure for 2 h.

### Analysis of the run products

The recovered Pt capsules were mounted in epoxy and polished for microanalysis. The phase assemblages of the run products were identified with an optical microscope and high-resolution elemental maps (Fig. 2) using an electron probe microanalyzer (EPMA) (JXA-8900 Superprobe; JEOL) at the Atmosphere and Ocean Research Institute, the University of Tokyo. The intensities of Si, Mg, Fe, Ca and Al were routinely determined by five wavelength-dispersive spectrometers (TAP, TAP, PETH, PETJ and LIFH, respectively) at an accelerating voltage of 15 kV, a probe current of 50 nA, and a focused beam. The intensity determination was conducted for 50 ms at intervals of 2 μm for the area of the entire of run product. A compositional image in backscattered electron mode was also compiled (Fig. 2). The major elements of the observed crystalline phases, quenched crystals and glass were analysed using EPMA with wavelength-dispersive spectrometers, an accelerating voltage of 15 kV, a probe current of 12 nA, a focused beam for crystals, 20 μm beam for the zone of quenched crystals and 50 μm beam for glasses. The ZAF correction was used.

### Confirmation of the attainment of equilibrium

Approach to equilibrium was verified using the following criteria. Phase proportions were calculated by mass balance calculations using linear least squares. The compositions of the zone of quenched crystals were included in the mass balance calculations because its thickness is commonly >10 μm (Fig. 2). Residual sums of squares (*∑R*^2^) less than 2.000 and the rough match between the calculated mass proportions and observed volume proportions on the polished surface of the run products is the primary evidence for the attainment of equilibrium. Olivine–melt, orthopyroxene-melt, clinopyroxene-melt and clinopyroxene-orthopyroxene Fe/Mg partition coefficients (*K*_D_) within the range 0.33±0.03 (ref. 39), 0.29±0.06 (ref. 38), 0.28±0.08 (ref. 38) and 1.09±0.14 (ref. 38; Supplementary Data 1), matching of the temperature reading of the thermocouple and the temperature at the sample position (1,258 °C), which was experimentally determined at 2.0 GPa and 1,250 °C using an enstatite–diopside mixed powder and the two-pyroxene geothermometer (within the error of geothermometer), the absence of chemical zoning in solid phases and the chemical homogeneity of glass and the zone of quenched crystals further confirm the attainment of equilibrium.

### Data availability

The data that support the findings of this study are available from the corresponding author upon request.

## Additional information

**How to cite this article:** Machida, S. *et al*. Petit-spot as definitive evidence for partial melting in the asthenosphere caused by CO_2_. *Nat. Commun.* **8,** 14302 doi: 10.1038/ncomms14302 (2017).

**Publisher's note:** Springer Nature remains neutral with regard to jurisdictional claims in published maps and institutional affiliations.

## Supplementary Material

Supplementary Data 1 A list of conditions and results of the melting experiments

Supplementary Data 2 Compositions of glasses and crystalline phases in experiments for KR04-08 D08-002 and YK06-05 6K#879-R3A

## Figures and Tables

**Table 1:**
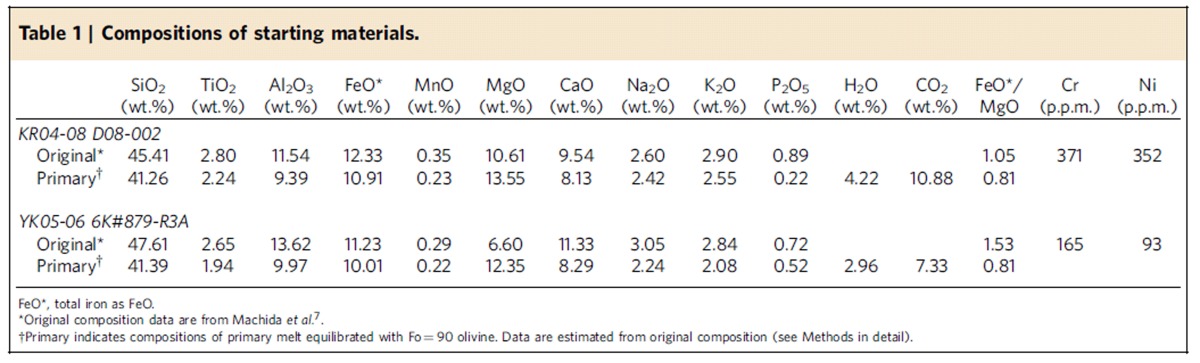
Compositions of starting materials.

*Reaction conditions:**1**/LiHMDS/**2**/[Pd(*η*^3^-C_3_H_5_)Cl]_2_/S-IPr·HCl=200/200/100/2.5/5; 0.1 M of ketone **1**; T=30^o^C; B/L and *dr* was determined by ^1^H NMR, *dr* is the ratio of (±)-(*syn,anti*)-**3**/other diastereoisomers; Isolated yield. †T=50 ^o^C. ‡Solvent=THF. §OBoc of **2** was replaced with OP(OEt)_2_. ||The yield was determined by ^1^H NMR.

## References

[b1] Hirschmann M. M. Partial melt in the oceanic low velocity zone. Phys. Earth Planet. Int. 179, 60–71 (2010).

[b2] Sifré D. . Electrical conductivity during incipient melting in the oceanic low-velocity zone. Nature 509, 81–85 (2014). 24784219 10.1038/nature13245 PMC4010644

[b3] Hirano N. . Volcanism in response to plate flexure. Science 313, 1426–1428 (2006). 16873612 10.1126/science.1128235

[b4] Obayashi M., Sugioka H., Yoshimitsu J. & Fukao Y. High temperature anomalies oceanward of subducting slabs at the 410-km discontinuity. Earth Planet. Sci. Lett. 243, 149–158 (2006).

[b5] Okumura S. & Hirano N. Carbon dioxide emission to Earth's surface by deep-sea volcanism. Geology 41, 1167–1170 (2013).

[b6] Hirano N., Kawamura K., Hattori M., Saito K. & Ogawa Y. A new type of intra-plate volcanism; young alkali basalts discovered from the subducting Pacific Plate, northern Japan Trench. Geophys. Res. Lett. 28, 2719–2722 (2001).

[b7] Machida S. . Petit-spot geology reveals melts in upper-most asthenosphere dragged by lithosphere. Earth Planet. Sci. Lett. 426, 267–279 (2015).

[b8] Hirano N., Koppers A. A. P., Takahashi A., Fujiwara T. & Nakanishi M. Seamounts, knolls and petit spot monogenetic volcanoes on the subducting Pacific Plate. Basin Res. 20, 543–553 (2008).

[b9] Hirano N. . Petit-spot lava fields off the central Chile trench induced by plate flexure. Geochem. J. 47, 249–257 (2013).

[b10] Taneja R. . ^40^Ar/^39^Ar geochronology and the paleoposition of Christmas Island (Australia), Northeast Indian Ocean. Gondwana Res. 28, 391–406 (2014).

[b11] Valentine G. A. & Hirano N. Mechanisms of low-flux intraplate volcanic fields—Basin and Range (North America) and northwest Pacific Ocean. Geology 38, 55–58 (2010).

[b12] Uenzelmann-Neben G., Schmidt D. N., Niessen F. & Stein R. Intraplate volcanism off South Greenland: caused by glacial rebound? Geophys. J. Int. 190, 1–7 (2012).

[b13] Buchs D. M. . Low-volume intraplate volcanism in the Early/Middle Jurassic Pacific basin documented by accreted sequences in Costa Rica. Geochem. Geophys. Geosyst. 14, 1552–1568 (2013).

[b14] Yamamoto J., Korenaga J., Hirano N. & Kagi H. Melt-rich lithosphere-asthenosphere boundary inferred from petit-spot volcanoes. Geology 42, 967–970 (2014).

[b15] Machida S., Hirano N. & Kimura J.-I. Evidence for recycled plate material in Pacific upper mantle unrelated to plumes. Geochim. Cosmochim. Acta 73, 3028–3037 (2009).

[b16] Médard E., McCammon C. A., Barr J. A. & Grove T. L. Oxygen fugacity, temperature reproducibility, and H_2_O contents of nominally anhydrous piston-cylinder experiments using graphite capsules. Am. Mineral 93, 1838–1844 (2008).

[b17] Green D. H. & Falloon T. J. in The Earth's Mantle ed. Jackson I. 311–378Cambridge University Press (1998).

[b18] Ghiorso M. S., Carmichael I. S. E., Rivers M. L. & Sack R. O. The Gibbs free-energy of mixing of natural silicate liquids — an expanded regular solution approximation for the calculation of magmatic intensive variables. Contrib. Mineral. Petrol. 84, 107–145 (1983).

[b19] Dalton C. A., Langmuir C. H. & Gale A. Geophysical and geochemical evidence for deep temperature variations beneath mid-ocean ridges. Science 344, 80–83 (2014). 24700855 10.1126/science.1249466

[b20] Kawakatsu H. . Seismic evidence for sharp lithosphere-asthenosphere boundaries of oceanic plates. Science 324, 449–502 (2009). 19390042 10.1126/science.1169499

[b21] Schmerr N. The Gutenberg discontinuity: melt at the lithosphere-asthenosphere boundary. Science 335, 1480–1483 (2012). 22442480 10.1126/science.1215433

[b22] Stein C. A. & Stein S. A model for the global variation in oceanic depth and heat flow with lithospheric age. Nature 359, 123–129 (1992).

[b23] Wallace M. E. & Green D. H. An experimental determination of primary carbonatite magma composition. Nature 335, 343–346 (1988).

[b24] Falloon T. J. & Green D. H. Solidus of carbonated fertile peridotite under fluid-saturated conditions. Geology 18, 195–199 (1990).

[b25] Sakamaki T. . Ponded melt at the boundary between the lithosphere and asthenosphere. Nat. Geosci. 6, 1041–1044 (2013).

[b26] Dasgupta R. . Carbon-dioxide-rich silicate melt in the Earth's upper mantle. Nature 493, 211–215 (2013). 23302861 10.1038/nature11731

[b27] Baba K., Abe N., Hirano N. & Ichiki M. Three-dimensional inversion analysis of seafloor magnetotelluirc data collected in the northwestern Pacific and implications for the source of petit-spot volcanoes. *5th International Symposium on Three-Dimensional Electromagnetics* Sapporo, Japan, May 7–9 (2013).

[b28] Harigane Y. . Direct evidence for upper mantle structure in the NW Pacific plate: microstructural analysis of a petit-spot peridotite xenolith. Earth Planet. Sci. Lett. 302, 194–202 (2011).

[b29] Nakanishi M., Tamaki K. & Kobayashi K. Mesozoic magnetic anomaly lineations and seafloor spreading history of the Northwestern Pacific. J. Geophys. Res. 94, 15437–15462 (1989).

[b30] Müller R. D., Sdrolias M., Gaina C. & Roest W. R. Age, spreading rates, and spreading asymmetry of the world's ocean crust. Geochem. Geophys. Geosyst. 9, Q04006 (2008).

[b31] Sobolev A. V. . The amount of recycled crust in source of mantle-derived melts. Science 316, 412–417 (2007). 17395795

[b32] Stolper E., Sherman S., Garcia M., Baker M. & Seaman C. Glass in the submarine section of the HSDP2 drill core, Hilo, Hawaii. Geochem. Geophys. Geosyst. 5, Q07G15 (2004).

[b33] Herzberg C. Identification of source lithology in the Hawaiian and Canary Islands: Implications for origins. J. Petrol. 52, 113–146 (2011).

[b34] Takahashi E. Origin of basaltic magmas-Implications from peridotite melting experiments and an olivine fractionation model. Bull. Volcanol. Soc. Jpn 30, S17–S40 (1986).

[b35] Machida S., Ishii T., Kimura J.-I., Awaji S. & Kato Y. Petrology and geochemistry of cross-chains in the Izu-Bonin back arc: Three mantle components with contributions of hydrous liquids from a deeply subducted slab. Geochem. Geophys. Geosyst. 9, Q05002 (2008).

[b36] Bose K. & Ganguly J. Quartz-coesite transition revisited: reversed experimental determination at 500-1200 °C and retrieved thermochemical properties. Am. Mineral. 80, 231–238 (1995).

[b37] Ohi S., Miyake A. & Yashima M. Stability field of the high-temperature orthorhombic phase in the enstatite-diopside system. Am. Mineral. 95, 1267–1275 (2010).

[b38] Putirka K. D. Thermometers and barometers for volcanic systems. Rev. Mineral. Geochem. 69, 61–120 (2008).

[b39] Toplis M. J. The thermodynamics of iron and magnesium partitioning between olivine and liquid: criteria for assessing and predicting equilibrium in natural and experimental systems. Contrib. Mineral. Petrol. 149, 22–39 (2005).

[b40] Green D. H. & Ringwood A. E. Mineralogy of peridotitic compositions under upper mantle conditions. Phys. Earth Planet. Int. 3, 359–371 (1970).

[b41] Hirschmann M. M. Mantle solidus: experimental constraints and the effects of peridotite composition. Geochem. Geophys. Geosyst. 1, 2000GC000070 (2000).

[b42] Green D. H. Experimental melting studies on a model upper mantle composition at high pressure under water-saturated and water-undersaturated conditions. Earth Planet. Sci. Lett. 19, 37–53 (1973).

[b43] Grove T. L., Chatterjee N., Parman S. W. & Médard E. The influence of H_2_O on mantle wedge melting. Earth Planet. Sci. Lett. 249, 74–89 (2006).

[b44] Till C. B., Grove T. L. & Withers A. C. The beginnings of hydrous mantle wedge melting. Contrib. Mineral. Petrol. 163, 669–688 (2012).

[b45] Green D. H., Hibberson W. O., Kovács I. & Rosenthal A. Water and its influence on the lithosphere–asthenosphere boundary. Nature 467, 448–451 (2013). 10.1038/nature09369 20865000

[b46] Falloon T. J. & Green D. H. The solidus of carbonated, fertile peridotite. Earth Planet. Sci. Lett. 94, 364–370 (1989).

[b47] Dasgupta R. & Hirschmann M. M. The deep carbon cycle and melting in Earth's interior. Earth Planet. Sci. Lett. 298, 1–13 (2010).

